# HIV-seroconversion among HIV-1 serodiscordant married couples in Tanzania: a cohort study

**DOI:** 10.1186/s12879-019-4151-8

**Published:** 2019-06-13

**Authors:** Soledad Colombe, James Beard, Baltazar Mtenga, Peter Lutonja, Julius Mngara, Claudia J. de Dood, Govert J. van Dam, Paul L. A. M. Corstjens, Samuel Kalluvya, Mark Urassa, Jim Todd, Jennifer A. Downs

**Affiliations:** 1 Center for Global Health, Department of Medicine, Weill Cornell Medicine, 402 East 67th Street, 2nd Floor, New York, NY 10065 USA; 2 Department of Population Health, London School of Hygiene and Tropical Medicine, London, UK; 3 National Institute for Medical Research, Mwanza Research Centre, Mwanza, Tanzania; 4 Department of Cell and Chemical Biology, Leiden University Medical Center, Leiden, Netherlands; 5 Department of Parasitology, Leiden University Medical Center, Leiden, Netherlands; 6 Department of Medicine, Bugando Medical Centre, Mwanza, Tanzania

**Keywords:** HIV, Modes of transmission, Heterosexual behavior

## Abstract

**Background:**

Heterosexual transmission is the main driver of the HIV epidemic in Tanzania. Only one estimate of the incidence rate of intra-marital HIV seroconversion in Tanzania has been reported and was derived from data collected between 1991 and 1995. Moreover, little is known about the specific risk factors for intra-marital seroconversion in Tanzania. Improved evidence around factors that increase the risk of HIV transmission to a serodiscordant spouse is needed to develop and improve evidence-based interventions. We sought to investigate the rate of intra-marital HIV seroconversion among HIV sero-discordant couples in Tanzania as well as its associated risk factors.

**Methods:**

We identified all HIV positive individuals in the TAZAMA HIV-serosurvey cohort and followed up their serodiscordant spouse from 2006 to 2016. The rate of seroconversion was analyzed by survival analysis using non-parametric regressions with exponential distribution.

**Results:**

We found 105 serodiscordant couples, 14 of which had a seroconverting spouse. The overall HIV-1 incidence rate among spouses of people with HIV-1 infection was 38.0 per 1000 person/years [22.5–64.1]. Notably, the HIV-1 incidence rate among HIV-1 seronegative male spouses was 6.7[0.9–47.5] per 1000 person/years, compared to 59.3 [34.4–102.1] per 1000 person/years among female spouses. Sex of the serodiscordant spouse was the only significant variable, even after adjusting for other variables (Hazard rate = 8.86[1.16–67.70], *p* = 0.036).

**Conclusions:**

Our study suggests that rates of HIV-1 seroconversion of sero-discordant partners are much higher within marriage than in the general population in Tanzania. The major risk factor for HIV-1 seroconversion is sex of the serodiscordant spouse, with female spouses being at very high risk of acquiring HIV infection. This suggests that future programs that target serodiscordant couples could be a novel and effective means of preventing HIV-1 transmission in Tanzania.

## Background

An estimated 790,000 new HIV infections occurred in Southern and Eastern Africa in 2016. Of these, 55,000 happened in Tanzania, where the prevalence of HIV is twice as high in women as in men [1]. Heterosexual transmission is the main driver of the HIV epidemic in Tanzania [2], with typical risk factors including presence of sexually transmitted infections (STI), *Schistosoma* spp. co-infection [3–5], low condom use, numerous sexual partners, and lack of male circumcision [6].

In 2011, the Tanzania HIV/AIDS and Malaria indicator survey showed that women and men who were married were more likely to be HIV infected compared to men and women that were never married [6]. Within marriage, additional factors associated with increased risk of HIV acquisition from a seropositive spouse include use of hormonal contraception [7], concurrent sexual partnerships [8], alcohol use [8], age discordancy [9], genital inflammation and discharge [10], non-antiretroviral (ART) use by the HIV-positive partner [11] and awareness of the partner’s status [12].

In addition, women in either long-term relationships or marriage are more at risk of acquiring HIV than men, likely due to an array of cultural, social, economic, and biological reasons [13]. In particular, male-to-female HIV transmission occurs with higher frequency than female-to-male transmission [14] and condoms are rarely used in intimate relationships in Tanzania [15]. Intimate partner violence and gender-based violence can also prevent women from protecting themselves against HIV [13] and women who experience intimate partner violence are as much as 1.5 times more likely to acquire HIV [13].

Only one estimate of the incidence rate of intra-marital HIV seroconversion in Tanzania has been reported and was derived from data collected between 1991 and 1995 [16]. Moreover, little is known about the specific risk factors for intra-marital seroconversion in Tanzania. Improved evidence around factors that increase the risk of HIV seroconversion within serodiscordant spouses is needed to develop and improve evidence-based interventions.

We sought to investigate the rate of intra-marital HIV-seroconversion within serodiscordant couples in Tanzania as well as its associated risk factors. We hypothesized that the rate of intra-marital HIV-seroconversion would be more than twice as high as the rate of HIV-seroconversion in the general population and that women would have higher rates of acquiring HIV than men.

## Methods

### Identification of HIV-1 infected individuals

Our study was conducted within the ongoing TAZAMA project, a community-based longitudinal open HIV-testing cohort in Kisesa, northwest Tanzania, which documents detailed demographic, sexual, and behavioral data and collects dried blood spots (DBS) approximately every 3 years from a population of ~ 30,000 individuals. Those wishing to know their HIV status may undergo voluntary HIV testing and counseling on the same day as collection of the DBS, and if tested positive, are referred to a treatment clinic. The HIV testing (sero-survey) is nested within a Demographic Surveillance System (DSS) which visits every household in the catchment area approximately every 9 months to document household members and relationships. Additional details have been previously described [17]. Details collected from both the DSS and sero-surveys included the start and end dates of sexual relationships with both the spouse with whom they lived and with external sexual partners and the frequency of sexual intercourse.

For this project, we identified all individuals whose DBS tested positive for HIV-1 or who were found to be HIV-1 positive at an HIV testing clinic within the TAZAMA cohort between 2006 and 2013. Throughout the rest of the methods we will refer to these individuals as “baseline individuals” for clarity and brevity.

### Identification of serodiscordant spouse and relationship time period

Through the DSS we identified all spouses of baseline individuals and obtained their HIV-1 test results from both the sero-surveys and from HIV tests at other clinics. We excluded couples that were never serodiscordant from the analysis, and couples for which the spouse had HIV-1 seroconverted more than 6 months after either partner reported the end of the relationship. For each couple, we determined the at-risk dates for HIV-1 seroconversion during which they reported being in a sexual relationship with a partner who was HIV-1 positive. We collected demographic and sexual behavior data from the first DSS or sero-survey following the start of the serodiscordant relationship. Sexual behavior data included the number of extra-marital partners, having sex with sex workers, and traveling men.

We used data from all sero-surveys until the last sero-survey with questions pertaining to the relationship time period. Seroconverters were defined as individuals who had been HIV-1 seronegative in one sero-survey and who were found to be HIV-1 seropositive in a subsequent sero-survey. All DBS available until the date of the spouse potential seroconversion were tested for *Schistosoma* circulating anodic antigen for both the baseline individual and his or her spouse.

### Follow-up

The follow-up period started either from the start of the relationship or from the first positive HIV result for the baseline individual. The follow-up period ended either at the spouse’s seroconversion date, or at the end of the relationship, or at the last sero-survey for which a spouse had an available HIV-1 test result and remained HIV-seronegative. The seroconversion date was approximated as the mid-point between the last negative DBS and the first positive test, either at a sero-survey or at another clinic.

### Laboratory testing

#### Dried blood spots

DBS were collected by finger prick onto a Whatman Protein Saver 903 card (GE Healthcare Bio-Sciences, Pittsburgh, PA). DBS cards were dried out of direct sunlight and sealed in a gas-impermeable zip bag with desiccant and humidity indicator. Cards were stored at the National Institute for Medical Research (NIMR) laboratory in Mwanza at − 20 °C.

#### HIV-1 testing

Diagnosis of HIV-1 infection was confirmed using a screening and subsequent confirmatory test, as recommended by national HIV guidelines, at each time point. These were: Uniform II Category III Ab test followed by Enzygnost test (sero-surveys 5 and 6), Uniform II Category IV Ab+Ag test followed by Enzygnost test (sero-survey 7), and Determine test followed by Unigold test (sero-survey 8). Samples that were negative at the screening test were reported as negative. Samples that were positive at the screening test were tested with the confirmatory test. If the confirmatory test was negative, the final result was reported as negative. If the confirmatory test was positive, the final result was reported as positive.

#### Schistosoma sp. testing

DBS were tested for schistosome for both HIV seronegative and seropositive individuals by Circulating Anodic Antigen (CAA) at Leiden University Medical Center by eluting whole blood from banked DBS collected during the sero-surveys and then concentrating the sample as previously described [18]. The CAA test is a genus-specific assay that detects a gut-associated antigen secreted into the host bloodstream by adult schistosome worms. The test does not differentiate between the *Schistosoma mansoni* and *haematobium* species present in the Kisesa area of Tanzania. A lower limit threshold of 2 pg CAA per mL of eluted blood was used for the assay. Thirty-five individuals had stored serum samples but no DBS samples available for testing and underwent serum CAA testing at NIMR with a lower limit threshold of 30 pg CAA per mL [19]. Samples scoring values above the threshold were designated positive for *Schistosoma* infection, and a person with schistosome positivity at any point in the time during the relationship was defined as having the infection.

### Statistical analysis

Analysis included all couples as described above. Binary variables were described as proportions and continuous variables were described using median and interquartile range. We assessed differences in baseline characteristics using Chi-square or Fisher’s exact test for proportions and the nonparametric equality test for medians.

A survival analysis was conducted to investigate the difference in HIV-1 seroconversion rates between spouse of a baseline individual by sex. The event of interest was defined as HIV-1 seroconversion. Data was censored at the end of the relationship or for loss to follow-up, defined as the last negative sero-survey at which the spouse provided a DBS. The Kaplan-Meyer method was used to compare time to seroconversion between the HIV-serodiscordant spouses of baseline individuals for males versus females. A parametric survival model with exponential distribution, adjusted for all significantly different baseline factors as well as biologically sound variables, was used to assess endpoint incidence difference by schistosome infection status. Time-dependent variables characterized at each sero-survey (such as number of extra-marital sex partners, frequency of sex, etc) were defined as representative of the time period following the sero-survey and are called “survey-dependent variables” for the rest of the manuscript.

Variables that were associated with failure at 10% significance were individually included into the model and model goodness-of-fit assessed through step-wise analysis. Based on the results of the analysis, a second analysis was performed after stratifying by sex of the serodiscordant spouse. All analyses were performed in STATA 14.1 (College Station, TX, USA). All results were expressed with 95% confidence intervals (CIs) and statistical significance was set at *P* < 0.05 (two-tailed).

We conducted a sensitivity analysis in which survey-dependent variables were defined as representative of the time period preceding the sero-survey results. Finally we conducted a second sensitivity analysis in which we excluded all couples for which the baseline individual was on ART.

### Ethics, consent and permissions

Ethical approval for retrospective and prospective analysis of these data was obtained from Bugando Medical Centre in Mwanza (BREC/001/04/2011), the National Institute for Medical Research in Dar es Salaam (NIMR/HQ/R.8a/Vol.IX/2446), and Weill Cornell Medicine in New York (1108011883). Study participants provided written informed consent during enrollment into the cohort study as per the approved procedures of the TAZAMA project, which included consent for future testing of DBS samples [17].

## Results

We identified 1439 baseline individuals who were found to be HIV-1 seropositive at a sero-survey between 2006 and 2013. Of these 554 had at least one spouse registered in the DSS after the time of the baseline individual’s first positive test. Among the 554, 289 had at least one spouse who had HIV-1 test results and 105/289 were serodiscordant couples between 2006 and 2016 who met criteria for inclusion in this analysis. From these serodiscordant couples, this yielded 368.8 years of total analysis time at risk and under observation.

63.8% (67/105) of couples had a male baseline individual and a female serodiscordant spouse. An overwhelming proportion of baseline individuals and spouses were of Sukuma ethnicity (97.1% (102/105) and 92.4% (97/105), respectively), Christian (83.8% (88/105) and 92.4% (87/105), respectively), and reported having only one spouse (87.6% (92/105) and 90.5% (95/105), respectively). 52.2% (48/92) of the baseline individuals were schistosome positive. 54.5% (55/101) of the serodiscordant spouses were schistosome positive. All couples were heterosexual. The demographics of the population are presented in Table 1 as a comparison between the 14 people who HIV-seroconverted during follow-up and the 91 people who did not. Serosurvey-dependent variables are presented in Table 2.

**Table 1:** Characteristics of the spouse, baseline individual and couple by spouse seroconversion status

Variable	Non-seroconverters *N* = 91	Seroconverters *N* = 14	*p*-value
Variables concerning the baseline individual			
Sex (Female)	40.7% (37/91)	7.1% (1/14)	0.016
Education (Received at least 1 year of formal schooling)	24.4% (22/90)	0.0% (0/14)	0.037
ART intake	12.1% (11/91)	0% (0/14)	0.353
Marital status (Polygamy)	13.2% (12/91)	7.1% (1/14)	1
Age in years at the start of the time period of interest	39[33–45]	44[37–53]	0.125
Schistosome CAA positivity	51.3% (41/80)	58.3% (7/12)	0.647
Variables concerning the serodiscordant spouse			
Sex (Female)	59.3% (54/91)	92.9% (13/14)	0.016
Education (Received at least 1 year of formal schooling)	42.9% (39/91)	21.4% (3/14)	0.037
Marital status (Polygamy)	12.1% (11/91)	0% (0/14)	0.353
Age in years at the start of the time period of interest	37[31–46]	35.5[32–46]	0.828
Male and circumcised	46.4% (13/28)	–	–
Schistosome CAA positivity	54.0% (47/87)	57.1% (8/14)	0.828
Variables concerning the couple			
Age difference between the baseline individual and his/her spouse	-3[−9;4]	−5[−8;-4]	0.246
Length of the time period of interest (in days)	1029 [691–1882]	1093.5[571–1150.5]	0.228

**Table 2:** Results of the univariable analysis for factors associated with HIV-1 seroconversion

Variable		Person-time (in years)	Number of events	Hazard ratio [95%CI]	*p*-value
Variables concerning the baseline individual					
Sex	Male	221.50	13	0.11[0.015–0.87]	0.036
	Female	149.43	1		
Education	Never attended school	294.91	14	0[0]^a^	0.992
	Ever attended school	71.01	0		
ART intake	No	318.72	14	0[0]^a^	0.992
	Yes	50.06	0		
Ln(CAA)^d^	–	–	–	1.18[0.93–1.49]	0.177
STI symptoms	No	291.31	11	1.03[0.29–3.68]	0.969
	Yes	77.46	3		
Schistosome CAA positivity	Negative	167.80	5	1.35[0.43–4.24]	0.611
	Positive	172.27	7		
Variables concerning the serodiscordant spouse					
Sex	Male	149.43	1	8.77[1.15–67.04]	0.036
	Female	221.50	13		
Education	Never attended school	234.99	11	0.48[0.13–1.72]	0.258
	Ever attended school	135.94	3		
Other risks for HIV^bd^	No	297.77	14	0[0]^a^	0.994
	Yes	71.00	0		
Risky sex behavior^c,d^	No	66.43	4	0[0]^a^	0.994
	Yes	19.15	0		
Ln(CAA) ^d^	–	–	–	1.11[0.85–1.44]	0.453
Number of extramarital partners^d^	None	303.95	14	0[0]^a^	0.994
	One or more	66.97	0		
STI Symptoms^d^	No	285.8	12	0.57[0.13–2.57]	0.468
	Yes	82.94	2		
Schistosome CAA positivity	Negative	152.33	6	1.03[0.36–2.98]	0.953
	Positive	196.75	8		
Variables concerning the couple					
Age difference between the baseline individual and his/her spouse in years	–	–	–	1.00[0.9991–1.001]	0.734
Sex frequency^d^	Less than once a month	37.22	1		
	Between once a month and once a week	134.64	6	1.66[0.20–13.78]	0.639
	More than once a week	166.50	5	1.12[0.13–9.57]	0.919

^a^No convergence of the model due to presence of zeros. No conclusion on the association between the variable and seroconversion can be made due to short person-time available. ART was still included in the final model stepwise analysis

^b^Other risks for HIV include incisions and transfusions

^c^Risky sex behaviors include having sex with women at bars or with traveling men

^d^Survey-dependent variables

14/105 (13.3%) partners HIV-1 seroconverted, and 13 of these were women. The overall HIV-1 incidence rate among spouses of people with HIV-1 infection was 38.0 per 1000 person/years [22.5–64.1]. Notably, the HIV-1 incidence rate among HIV-1 seronegative male spouse was 6.7[0.9–47.5] per 1000 person/years, compared to 59.3 [34.4–102.1] per 1000 person/years among female spouse.

After univariable analysis, sex of the serodiscordant partner was the only variable that was significantly associated with HIV-1 seroconversion. HIV-1 uninfected female spouses of HIV-1 infected male baseline individuals were found to have higher incidence rates of seroconversion than HIV-1 uninfected male spouses of HIV-1 infected female baseline individuals (Hazard ratio (HR) = 8.77, *p* = 0.036). None of the spouses of baseline individuals on ART or with formal education seroconverted. None of the serodiscordant spouses in a polygamous marriage or with reported extra-marital partners acquired HIV. Results of the univariable analyses are presented in Table 2.

**Fig. 1:**
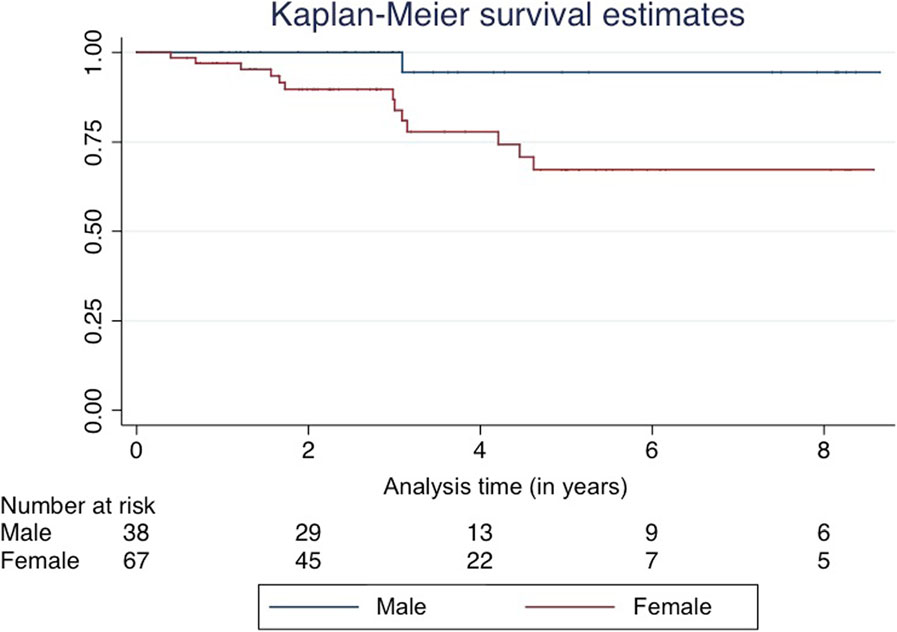
Kaplan-Meier survival estimates for seroconversion by sex of the spouse. The curve represents the risk of seroconverting over time by sex of the serodiscordant spouse

After stepwise multivariable analysis, sex of the serodiscordant partner was the only variable that yielded a best of fit model. Female spouses thus had a rate of seroconversion 8.77[1.15–67.04] times higher than male spouses (*p* = 0.036). The Kaplan-Meyer survival curves by spouse sex are presented in Fig. 1.

After running the sensitivity analyses, when survey-dependent variables were defined as representative of the time period preceding the sero-survey results, the hazard ratio for female spouses was 8.86[1.16–67.70], *p* = 0.036. When excluding all couples on ART from the analysis, the hazard ratio for female spouses was still 8.89[1.16–67.92], *p* = 0.035. Finally, when stratifying by sex, none of the variables were significantly associated with seroconversion.

## Discussion

In this in-depth study of a community of approximately 30,000 individuals, the intra-marriage HIV-incidence in our study population was overall 19 times the general national HIV-incidence [1]. This effect was largely due to women being highly susceptible to incident HIV infection, yielding an incidence of 60 seroconversions per 1000 person-years in women and only 7 per 1000 person-years in men. This is a greater than eight-fold increase in HIV acquisition in women as compared to men, and suggests that intra-marriage seroconversion in serodiscordant couples deserves more attention in Tanzania, and that disproportionate transmission from men to women, particularly in the absence of female-controlled HIV-prevention measures, may continue to push the HIV epidemic towards female predominance.

Only one other estimate of the intra-marriage HIV- incidence in Tanzania has been published, reporting a rate of HIV seroconversion among serodiscordant couples of 75(28–163) per 1000 person-years between 1991 and 1995, with the rate of seroconversion for women only being twice as high as the rate of seroconversion for men [16]. The difference between the prior and current findings reflects the large decrease in HIV incidence in Tanzania over the past 30 years as well as changes in drivers of the epidemic, such as changes in sexual behavior and women’s susceptibility. In other countries in Sub-Saharan Africa, reported HIV incidence in HIV-serodiscordant couples concord with our results, although varies widely (from 20 to 118 per 1000 person-years) [20–22]. Of note, these studies reported rates in serodiscordant negative females less than twice as high as rates in serodiscordant negative males [20].

Although uptake of ART among HIV-serodiscordant couples has been recommended where feasible [23], it does not remove the need for behavioral interventions to reduce HIV acquisition from outside partners [12]. Serodiscordance within a couple may lead to increased outside-partnership sex and increased risk of outside transmission [12]. Tanzania has not focused its efforts on serodiscordant couples yet and these results suggest that future programs that target serodiscordant couples may be an effective way to prevent HIV-1 transmission in Tanzania. Couples’ voluntary HIV counseling and testing significantly reduces HIV transmission in discordant couples and has been shown to increase a couple’s knowledge about HIV serodiscordance and prevention behaviors [21]. In the light of the shift to test-and-treat strategies, couples’ targeted-strategies for increasing uptake of ART could be a key tool to achieve the goal of eliminating HIV transmission, and this should include specific HIV prevention messages to the HIV negative partner.

Surprisingly, the usual risk factors for HIV-1 seroconversion within marriage, such as sex frequency, count of extra-marital sex partners and age difference with the spouse, were not significant within our cohort [24]. It is possible that this was due to the higher age of our study participants than has been observed in most other serodiscordant couple studies [4, 16]. In these older adults, traditional HIV risk factors may be less important. Our results are consistent with the well-described finding that, per sex act, women are indeed more at risk of HIV-1 infection than men [25], likely due to a larger surface area of the vagina and the ability of the virus to pass easily through the cells of the vaginal lining. Too few partners were on ART to assess the role of ART on seroconversion in our study, although the finding that no partners of baseline individuals on ART seroconverted is consistent with other studies that showed lower incidence of HIV when HIV positive partner was on ART.

Our study supports findings of a large independent serodiscordant couples’ study in Zambia. Our estimate of the role of schistosome infection in the transmitting partner on HIV-1 incidence (HR = 1.35, *p* = 0.6) is of similar magnitude of that found in the much larger Zambia study [4] (adjusted HR between 1.4 and 1.8, *p* < 0.005) and shows a directional trend towards an increased transmission of HIV-1 from baseline individuals infected with *Schistosoma* spp.. Schistosome infection could lead to genital inflammation and exposure to blood or higher genital tract HIV-1 RNA viral loads [26, 27]. *Schistosoma* spp. leads to higher HIV-1 RNA plasma viral load as well [5], which is highly predictive of transmission to sexual partners [28]. Even though our project was located in a community cohort of 30,000 people, our sample size of discordant couples provided only enough power (80%) to detect significance for a hazard ratio of 3.7 or above, far higher than the hazard ratio estimates obtained by this or the Zambia study. Our study similarly may have lacked power to document an effect of schistosome status on HIV acquisition in the receiving partner, which has previously been documented in other studies [5, 29].

Our results are to be interpreted in light of some limitations. We were unable to test for viral loads, or additional immunologic markers that might provide insight into the reasons for our observations, due to insufficient quantity of blood in DBS. We were also unable to perform phylogenetic analyses that would have permitted determination of whether the HIV-seroconverting partner had been infected from a partner outside of the marriage. Partners might also under or overestimate the number of extra-marital sexual partners based on their gender [30]. Further studies with larger numbers of seroconverters should be undertaken, which should include better assessment of loss to follow up among the HIV negative marital partners of PLHIV. Despite those limitations, our finding that all sensitivity analyses gave the same results strengthens confidence in the quality and accuracy of our analysis.

## Conclusions

In conclusion, our study suggests that rates of HIV-1 seroconversion are much higher within marriage than in the general population in Tanzania. The major risk factor for HIV-1 seroconversion is sex of the serodiscordant spouse, with female spouses being at very high risk of HIV-seroconverting.

This suggests that future programs that target serodiscordant couples, regardless of source of infection, could be a novel and effective means of preventing HIV-1 transmission in Tanzania. In addition, future studies should explore the source of HIV acquisition in order to focus HIV prevention efforts on specific risk groups.

## Data Availability

Data are available to researchers who meet the criteria for access to confidential data from the TAZAMA project, upon request. The TAZAMA data follow the INDEPTH Policies, which requires information and signatures before obtaining the data. Interested researchers should contact John Changalucha at jchangalucha@yahoo.com.

## Abbreviations

AIDS: Acquired Immune Deficiency Syndrome
ART: Antiretroviral Therapy
CAA: Circulating Anodic Antigen
DBS: Dried Blood Spot
DSS: Demographic Surveillance System
HIV: Human Immunodeficiency Virus
HR: Hazard Ratio
NIMR: National Institute for Medical Research
RNA: Ribonucleic acid
STI: Sexually Transmitted Infection
